# An incomplete picture: data limitations in costed National Action Plans for Health Security (NAPHS)

**DOI:** 10.1136/bmjgh-2023-014067

**Published:** 2024-02-21

**Authors:** Ciara M Weets, Stephanie Eaneff, Rebecca Katz

**Affiliations:** 1 Georgetown University Center for Global Health Science and Security, Washington, District of Columbia, USA

**Keywords:** Health policy

**SUMMARY BOX**

- Reliable, evidence-informed cost estimates are critical to inform financing decisions in pandemic preparedness and response (PPR).
- Over 80 countries have completed National Action Plans for Health Security (NAPHS), which contain national-level plans to address known gaps in health security.
- In some cases, these plans are developed and/or published alongside detailed costing information to inform budgetary decisions.
- Our analysis found that, as of September 2023, only 11% (9/82) of countries that had completed a NAPHS included costed line-item data (e.g. budget information) within published materials.
- The shortcomings of NAPHS data accessibility and reliability highlight the importance of transparency and enhanced data sharing throughout the NAPHS process.
- The sharing and aggregation of data from costed NAPHS would allow for funding gaps to be identified, regional trends to be characterised, advocacy efforts to be concentrated and financing requirements to be clarified.

Transparent, reliable estimates of cost are critical to inform discussions about the future of financing for pandemic preparedness and response (PPR). Estimates of global investment requirements for national-level capacity building for health security under the International Health Regulations (IHR) range from US$107.2 billion to US$204 billion^1^ over a 5-year period, but limited information is available on those cost requirements disaggregated at the national level. Reliable data, including both global and national-level estimates, are critical to inform ongoing financing decisions, including those at the World Bank’s Pandemic Fund, where the first round of funding has already been released and additional calls for funding are expected shortly.

Since 2016, the WHO has worked with countries to assess their existing health security capacity, and to follow those assessments with national plans and budgets to address any identified gaps. As part of this process, countries first participate in a voluntary, external assessment—a Joint External Evaluation (JEE). The assessment is conducted through a partnership between teams of in-country and external subject area experts to assess health system competencies and provide recommendations to enhance preparedness.^2^ On the basis of these assessments, each country then works with WHO to complete a costed National Action Plan for Health Security (NAPHS), which reflects plans and associated cost estimates that address the gaps identified by the JEE assessment.^3^ Many, but not all, countries share these costed plans publicly via WHO’s online NAPHS portal.^4^

However, most published costed NAPHS data are not comprehensive. As of September 2023, based on data from WHO’s online NAPHS portal, 119 countries have completed one or more JEE and 82 have completed one or more NAPHS; however, only 14/82 (17%) have published any NAPHS via WHO’s web platform.^4^ Of those completed, only 9/82 (11%) included costed line-item data (eg, budget information) within their published materials. No high-income countries, according to World Bank’s income designation, have published any costed line-item data. None of the published NAPHS on the WHO portal were available for download in a machine-readable format, for example, a comma separated value file (.csv) or an Excel file (.xls), necessitating a significant amount of human labour to make these data accessible and actionable.

Moreover, costed NAPHS data that *are* publicly available paint an incomplete picture of health security investment requirements. In particular, across a number of countries, cost estimates for certain high-cost items are excluded from reporting and calculations. Said another way, these costs are treated as ‘zero cost’ in final aggregate calculations, despite many of them being associated with activities that require substantial financial commitments. In the majority of cases, these zero cost activities are reported without explanation or additional documentation. Notable zero-cost items include activities such as ‘Develop e-surveillance tools at national, intermediate and local levels of the surveillance system to support all aspects of the surveillance process…’^5^ and ‘Procure and supply yellow fever vaccines and other consumables…’.^6^ Such examples of missing data exist across numerous costed NAPHS, and have substantial implications for the use of these data without more detailed considerations of the underlying data sets and their limitations. Moreover, published line-item cost data reflect known limitations of both the NAPHS and JEE process, including a primary focus on national-level activities (vs subnational) and potential underestimates of personnel and capital investments, both of which also depend on the version of the assessment used to generate cost estimates.^7^ Put simply, using published data from NAPHS documents with zero-ed out or otherwise incomplete costs, without somehow accounting for these costs, will lead to underestimates of the true funds required for PPR capacity building.

The NAPHS framework and associated data can provide critical information to evaluate, plan, and budget for health security and health system strengthening, yet publicly available data are limited. These data are needed to develop comprehensive plans that reliably estimate financing requirements for key aspects of PPR, including capital investments and personnel costs. Together, national plans and their associated data are valuable sources of information for funders, advocates, implementers, and countries looking to model best practices and to help ensure that funds are prioritised. However, in all cases, the usefulness of these plans and cost estimates depends on a robust understanding of which investments are, and are not, being prioritised and costed—this requires transparency regarding underlying data and methods. Moreover, representative data are needed across WHO regions and country income levels.

Based on these findings, we advocate for countries to publicly share their costed NAPHS data in analysis-friendly formats, to increase transparency and promote international collaboration on global health preparedness and security. In the future, such costing data could be used to inform the development and improvement of existing health budgeting tools, for instance, by creating a centralised database of interventions and associated budget examples, building on similar work done to estimate the cost of IHR implementation.^8^ Moreover, analysis of existing data would allow gaps in funding to be identified, trends within countries and regions to be characterised, advocacy efforts to be concentrated, and financing requirements across sectors and disease verticals to be clarified.

## Data Availability

The data referenced in this publication, as well as the code used to complete analysis and generate figures, are publicly available at https://github.com/cghss/NAPHS-data/tree/main/global-summary.
